# Recurrence of Basal Cell Carcinoma Treated with Surgical Excision and Histopathological Analysis with Frozen Section Technique with Complete Margin Control (CMC-FS): A 15-Year Experience of a Reference Center

**DOI:** 10.3390/cancers15153840

**Published:** 2023-07-28

**Authors:** Alessandra Di Maria, Gianmaria Barone, Vanessa Ferraro, Costanza Tredici, Sofia Manara, Camilla De Carlo, Alessandro Gaeta, Filippo Confalonieri

**Affiliations:** 1 Department of Ophthalmology, IRCCS Humanitas Research Hospital, 20089 Milan, Italy; alessandra.di_maria@humanitas.it (A.D.M.); gianmaria.barone@humanitas.it (G.B.); costanza.tredici@humanitas.it (C.T.); filippo.confalonieri@humanitas.it (F.C.); 2 Department of Biomedical Sciences, Humanitas University, 20090 Milan, Italy; sofia.manara@humanitas.it (S.M.); camilla.de_carlo@humanitas.it (C.D.C.); 3 Department of Pathology, IRCCS Humanitas Research Hospital, 20089 Milan, Italy; 4 Department of Internal Medicine and Medical Specialties (DIMI), Università di Genova, 16132 Genova, Italy; alessandrogaeta01@gmail.com

**Keywords:** basal cell carcinoma, CMC-FS frozen section analysis, eyelid carcinoma, controlled excision via frozen section, recurrent basal cell carcinoma

## Abstract

**Simple Summary:**

This research reports on the efficacy in terms of oncological radicality of upper-third, facial basal cell carcinomas treated with excision with the frozen section technique with complete margin control, comparing the recurrence rate to the gold standard Mohs micrographic surgery. Our observations revealed that the implementation of the complete margin control frozen section technique exhibited an eradication rate and subsequent recurrence rate comparable to that of the conventional Mohs micrographic surgery technique, widely regarded as the gold standard in the existing literature. This intriguing finding highlights the paradox that a faster and more expeditious technique can demonstrate a non-inferior profile when compared to the established benchmark in the field.

**Abstract:**

Background: Basal cell carcinoma (BCC) is the most common type of eyelid malignancy and it is considered to be dangerous due to its proximity to functionally essential organs. Early diagnosis and complete excision of the primary lesion are crucial to prevent infiltration and metastasis. The study aims to evaluate the extent of recurrence in subjects affected by BCC of the upper third of the face treated with surgical eradication and the frozen section technique with complete margin control (CMC-FS), in comparison with the gold standard Mohs micrographic surgery (MMS). Materials and Methods: The study included 111 patients with 111 biopsy-proven eyelid BCCs. On clinical examination, all lesions were removed with 2 mm margins clinically free of neoplasm. Prior to reconstruction, CMC-FS analysis of all surgical margins was performed on each tumor for histopathologic confirmation. Subsequently, all margins were presented for the permanent paraffin sections. Results: There were 69 primary carcinomas and 42 secondary carcinomas among the 111 samples. No recurrence occurred in 109 tumors followed-up for at least 5 years, with a total recurrence rate of 1.8%. The median time between lesion excision and diagnosis of recurrence was 20 months. Conclusions: at 5-year follow-up, CMC-FS guided excision of BCCs of the eyelids resulted in recurrence rates equivalent to MMS. Intraoperative microscopic control of all margins reduced the recurrence rate of the upper third of the facial BCCs, correlating with easier reconstruction with better esthetic and functional outcome.

## 1. Introduction

Basal cell carcinoma (BCC) is a neoplastic transformation of the basal cells of the skin, which can proliferate and penetrate the underlying dermis [1]. Eyelid malignancies account for over 90% of all ocular malignancies, with BCC accounting for 90% of all eyelid malignancies. The typical occurrence of this condition is in areas of the body that are exposed to the sun. Around 80% of cases tend to develop on the head and neck, while approximately 15% are observed on the trunk and arms [2,3]. Around 5% to 10% of all skin neoplasms are localized specifically on the eyelid [4,5]. Due to its proximity to functionally essential organs such as the eye sockets, sinuses, and brain, eyelid BCC is considered dangerous [6]. The estimated lifetime risk for developing basal cell carcinoma (BCC) in the general population is around 30%. Moreover, approximately 95% of BCC cases occur in individuals between the ages of 40 and 79 years [7]. Individuals with fair skin who have a history of chronic sun exposure and subsequent skin damage are at a higher risk of developing skin neoplasms, including basal cell carcinoma.

Periocular basal cell carcinoma (BCC) typically exhibits the following distribution across the eyelid regions: the lower eyelid accounts for approximately 43% of cases, followed by the medial canthus (26%), upper lid (12%), and lateral canthus (8%) [8,9,10]. While basal cell carcinomas (BCCs) rarely metastasize and tend to grow slowly, it is important to seek timely treatment. If treatment is delayed, BCCs can continue to grow and potentially infiltrate nearby structures such as the eye sockets, nose, sinuses, and base of the skull. Therefore, early detection and prompt treatment are crucial to prevent further progression and potential complications [11]. To minimize morbidity and mortality associated with skin neoplasms, particularly to improve the chances of a complete cure, early diagnosis and complete surgical removal of the primary lesion are essential. By identifying the condition at an early stage and ensuring the thorough excision of the cancerous growth, the likelihood of successful treatment and long-term survival can be significantly increased [12]. Recurrent BCCs, in fact, behave biologically more aggressively and have worse prognoses than primary tumors [13]. Recurrences account for 84% of periocular BCCs that penetrate the orbit and recurrent BCCs have a 50% risk of recurrence [14,15,16]. The goal of treatment should be total elimination of the tumor. The treatment of choice is surgical eradication.

Radiation therapy, electrodrying and curettage, conventional excision, Mohs micrographic surgery (MMS), frozen section-controlled excision (FSC), photodynamic therapy and other medical treatments are the therapeutic tools for periocular skin neoplasms [17]. The only surgical procedures with microscopic control of the intraoperative margin in order to reduce recurrence rates are MMS and FSC excisions [18,19,20,21].

MMS is a specialized technique commonly employed for the treatment of different types of skin malignancies. One of its primary advantages is the preservation of uninvolved tissue, which can potentially simplify the process of reconstruction after tumor removal. MMS is particularly regarded as an effective approach for the excision of BCC. By meticulously examining the excised tissue layers under a microscope during the procedure, MMS allows for precise removal of the cancerous cells while minimizing damage to surrounding healthy tissue. This technique enhances the chances of complete tumor removal and reduces the likelihood of recurrence, thus contributing to favorable treatment outcomes for BCC [4,22,23]. During Mohs micrographic surgery, the tumor-containing tissue is removed in small, saucer-like sections. To maintain the proper orientation of the tissue, a color-coding system is used. The excised tissue is inked for reference and relaxed incisions are made within the specimen. These incisions help flatten the three-dimensional tissue onto a two-dimensional slide, allowing for better examination under a microscope. Additionally, these incisions create continuous outer margins, which help ensure complete tumor removal and minimize the chance of leaving any cancerous cells behind. This meticulous process allows for precise evaluation of the surgical margins and helps achieve the highest possible cure rates for skin malignancies [24]. Traditionally, blunt sections are taken, allowing for visualization of the epidermis, dermis, and subcutaneous tissue. 

If a margin is found to be positive for tumor cells, additional fine excisions can be performed specifically in the identified area using the mapping technique. This targeted approach allows for the removal of the remaining tumor cells while maximizing the preservation of uninvolved tissue. However, it’s important to note that this method can be labor-intensive due to the meticulous nature of the procedure. CMC-FS excision of periocular skin tumors has been shown to be a reliable therapeutic strategy for the management of BCC as an alternative to MMS [4]. 

The visible areas of the tumor are carefully delineated in the standard frozen section technique, and a 3–4 mm border of normal tissue surrounding the tumor is inked. The entire tumor block is surgically removed, together with the specified boundary of normal tissue, and sent for histopathologic assessment. If any surgical margin is discovered to have tumor cells, an extra 2 to 4 mm of tissue is harvested from the neighboring region. This procedure is repeated until all surgical margins are tumor-free. Suture marking, tissue inking, written diagrams, accurate mapping, and clear communication between the surgeon and pathologist are employed to preserve orientation in lid or periocular tissue. Reconstruction is normally conducted as soon as a histological test confirms the presence of clean margins, showing the absence of tumor cells [25].

During frozen section control, when examining surgical specimens, there are two approaches for processing margin sections: perpendicular and en face (or parallel) to the surgical margin. The conventional method involves making perpendicular sections during tumor excision. These sections allow for the examination of the tumor’s morphological characteristics in the frozen material and help determine if it extends to the margin. Additionally, they provide information about the distance between the tumor and the margin if the tumor is not present at the margin. However, a drawback of the perpendicular slices method is that it does not examine the entire margin. Since pathologists typically “bread” the tissue into slices approximately 1–2 mm thick, margins in certain areas of the sample may not be assessed unless the fabric block is trimmed.

On the other hand, en face sectioning provides the opportunity to examine the entire margin using a relatively smaller number of sections. This method involves making thin sections to minimize the risk of misinterpreting the histological margin. By utilizing en face sectioning, a comprehensive assessment of the entire margin can be achieved while maintaining clarity in the histopathological evaluation. If the en face method is used, the fabric can be incorporated with the edge facing up or down. The advantage of the margin down technique is the reduced requirement for extensive levels during evaluation. However, the drawback is the increased difficulty in accurately orienting these specimens. On the other hand, margin down allows for a comprehensive section from the margin nearest to the tumor. In certain cases, en face sectioning may present challenges in obtaining smooth and complete tissue slices, leading to areas of the margin being unavailable for evaluation. If the initial section is incomplete, additional cropping may be necessary to obtain a complete image of the margins. A further problem is that en face sectioning does not allow for the determination of the distance between the tumor and the margin. Even if the margin is negative, the tumor could be negative a few micrometers away. Therefore, in cases where en face sectioning is utilized during controlled excision of frozen sections, it is important to note that even if a 4 mm margin appears clear, it is still not considered adequate. It is advisable to maintain a clear clinical margin to reduce the risk of having an insufficiently small histological margin.

The CMC-FS technique incorporates various essential components for tumor excision. These include the utilization of drawings to accurately map the surgical specimen’s margins, the application of tissue inking, the use of thin and relatively small sections, clinical assessment of the margins, and en face sectioning with margins in an upward direction. This technique can effectively achieve oncological radicality similar to that of the Mohs surgeon. 

Many investigators have reported a follow-up duration of 5 years with MMS; however, most CMC-FS excision studies have had a relatively short mean follow-up time (≤5 years) [5,25,26,27,28,29,30,31,32,33,34,35,36,37,38]. The aim of our study is to report the efficacy in terms of oncological radicality of upper third facial BCCs treated with excision and CMC-FS, comparing the recurrence rate to the gold standard MMS. 

## 2. Materials and Methods

Patients were treated at the Oculoplastic Unit, Department of Ophthalmology, IRCCS Humanitas Research Hospital, Rozzano, Milan, Italy from January 2003 to December 2018. The study was compliant with the Health Insurance Portability and Liability Act and in accordance with the principles outlined in the Declaration of Helsinki (2013). The study was approved by the Institutional Council.

A retrospective review of the medical records of 272 lesions operated on for clinical suspicion of BCC was collected. Patients with primary and secondary upper-third facial BCCs treated with surgical excision and CMC-FS were included in the study. Primary conjunctival tumors, lesions that migrated to nearby lymph nodes, and individuals whose lesion received a biopsy-proven diagnosis other than BCC were excluded. Due to failure to complete a minimum of 5 years of clinical follow-up, 44 patients were excluded. All excised tissue samples were analyzed by experienced pathologists. Each lesion was treated by the Oculoplastic and Reconstructive Surgery Unit of the Ophthalmology Department of the IRCCS Humanitas Research Hospital, Rozzano, Milan, Italy.

All patients underwent the surgical procedure described here: depending on the patient’s comorbidities, on the planned restorative treatment and on the extent of the lesion, excision was performed under general anesthesia or local anesthesia with sedoanalgesia. Before the administration of anesthesia, the resection margins were outlined. Below and around the region designated for excision, a 50/50 combination of 0.50% bupivacaine and 2% mepivacaine with epinephrine was given. At the clinical evaluation, all the tumors were removed with a minimum of 2 mm margin free from neoplasia and the samples were sent to the pathology department oriented on a rigid support and/or with notches. To rule out tumor invasion at the intraoperative excision margin, CMC-FS excision was performed. Two main categories of specimens can be distinguished: wedge eyelid skin excision biopsy and simple ellipsoidal skin excision biopsy.

**Figure 1:**
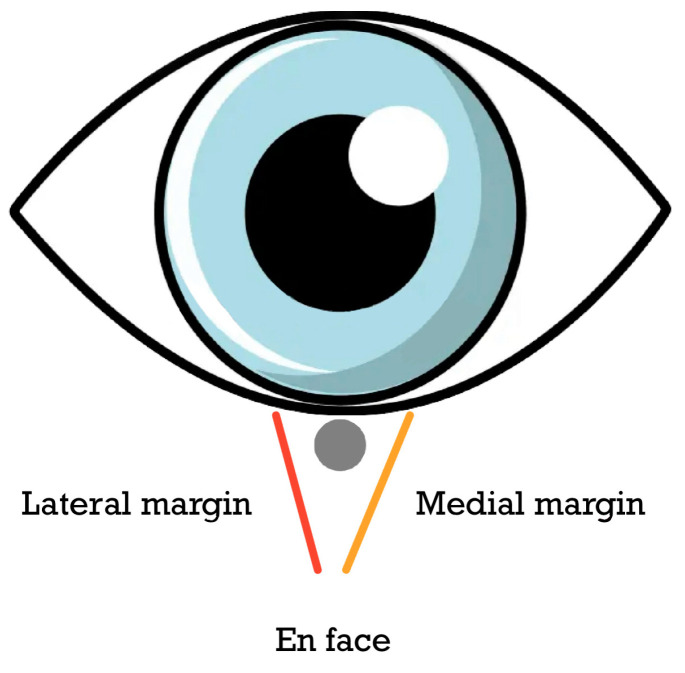
En face technique.

**Figure 2:**
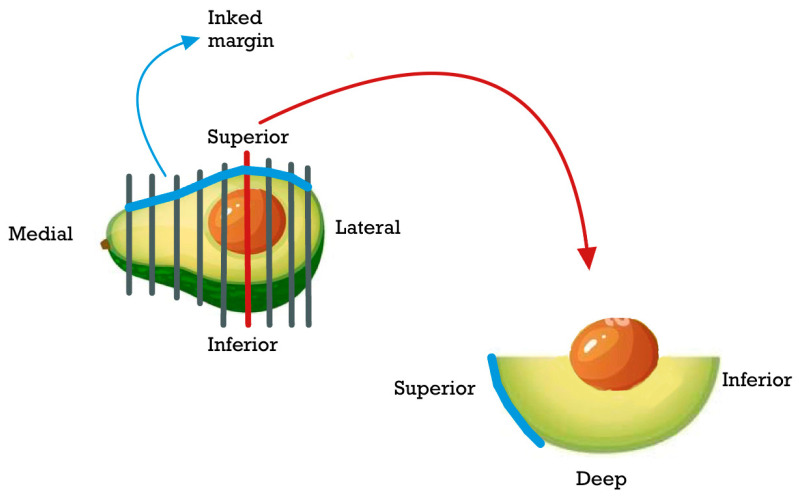
Color inking technique.

The complete assessment of the margins was performed in the first case with a few full-thickness en face sections (Figure 1). In the second case however, after double color inking, it was carried out with serialized sections in the loaf technique, including all the superficial and deep margins (Figure 2). In both cases, margins were evaluated on frozen sections. Sections were placed in mounting medium, frozen using liquid nitrogen, sectioned at approximately 5 µm, and then stained with hematoxylin-eosin.

**Figure 3:**
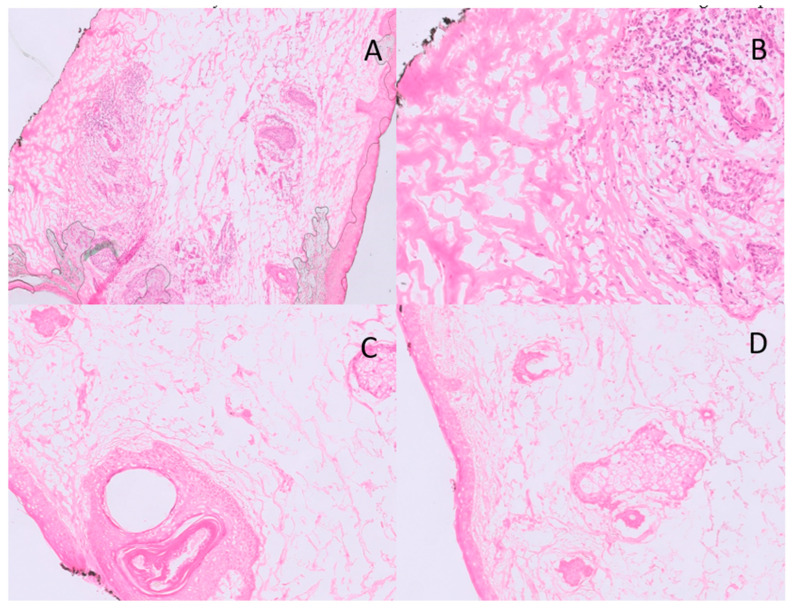
An example of BCC positive margin at low (**A**) and high (**B**) power magnification (H&E); in the hypodermis, small nests of BCC are visible, closely related to an inflammatory focus. An example of BCC negative margin at low- (**C**) and high-power (**D**) magnification (40×).

After each margin evaluation, the pathologist called the surgeon in the operating room by phone to communicate the diagnosis. A second sample was taken from the area where residual tumor was visible when a sample was found to have tumor cells after histopathologic analysis. Until clean margins were achieved, fresh tissue from the excision was delivered to the pathology laboratory. Only when the histopathological analysis no longer found tumor cells in any margin the oculoplastic surgeon could perform the reconstruction (Figure 3). The diagnosis of the frozen slices was then confirmed by sending all samples for long-term paraffin sections. Through this mixed approach, precise microscopic control of all margins can be achieved with more effective preservation of normal tissue and reduced sacrifice of healthy tissue.

Patients were followed up for at least 5 years, after the first year after the removal of the BCCs a clinical evaluation was performed once a year. All patients who did not attend their annual follow-up were telephoned and re-evaluated. They underwent an ophthalmological examination and were evaluated for any potential type of recurrence. When a potential recurrence was detected during clinical examination, the patient underwent a biopsy to establish a histopathologic diagnosis. Various details were documented for each patient, including age at the initial visit, gender, previous treatments received, anatomical location of the lesion, maximum diameter of the tumor, number of margins analyzed, number of resections needed to achieve clear margins, diagnoses from both frozen section and permanent paraffin section evaluations, as well as any instances of recurrence following treatment. New-onset BCCs were classified as primary lesions, whereas previously treated and recurrent BCCs were classified as secondary lesions. Recurrence was defined as recurrence of the lesion at the same anatomical site at the follow-up clinic evaluation.

BCC recurrence rate and predictive power of CMC-FS were compared with those reported in the literature for MMS.

Statistical analysis was conducted to examine the associations between categorical parameters using *t*-tests. The correlations between continuous variables were evaluated using the Pearson correlation coefficient and linear regression. The data were analyzed using STATA, a specialized statistical software for data science. A significance level of 0.05 was employed to determine statistically significant differences between groups.

## 3. Results

Primary and secondary upper third facial BCCs recurrence after surgical removal and CMC-FS analysis were evaluated with a 5-year follow-up. A total of 111 biopsy confirmed BCCs were enrolled.

The characteristics of the sample are summarized in Table 1.

The mean age at diagnosis was 69 ± 11 years. All patients were Caucasian, 47 (42.3%) of them were men and 64 (57.7%) were women. The considered BCCs had a mean maximum lesion diameter of 0.88 ± 0.99 cm (range 0.3 cm to 1.7 cm). Regarding the anatomical location of BCC, 81 tumors (73%) were located on the lower eyelid, 19 (17.1%) on the upper eyelid, 8 (7.2%) on the medial canthus, and 3 (2.7%) on the lateral canthus.

Of the 111 lid BCCs, 71 (64.0%) required only one CMC-FS analysis before all samples tested negative for tumor cells, 24 (21.6%) required two phases, 12 (10.8%) three phases, 3 (2.7%) four and only 1 (0.9%) five phases. Patients were followed up for a mean of 65.7 ± 7.5 months (range 60–100) and the overall recurrence rate was 1.8% (2 out of 111 lesions). Out of 111 patients, 42 (37.8%) had secondary BCCs and they were treated with the same technique as they were primary tumors. The recurrence rate after this excision was higher in the secondary BCC group (2.4%, 1 of 42 lesions), than in primary BCC group (1.4%, 1 of 69 lesions) but the difference was not statistically significant (*p* = 0.37). In the two patients with recurrent tumors, the original tumor size was 0.9 cm and 1.4 cm and both carcinomas involved the lid margin. At diagnosis, the first patient had a primary BCC on the low lid and the second had a secondary BCC on the upper lid. The first tumor was removed with a single en bloc excision and showed no evidence of malignant cells on histopathologic examination of the tumor margins. The second tumor required five stages of resection before all margins were negative for tumor cells. The median time between lesion excision and diagnosis of recurrence was 20 months (18 and 22 months, respectively). Both recurrences were treated with a new CMC-FS excision after the diagnosis confirmed by biopsy. One patient was followed up for 3 years after recurrence and had no evidence of recurrence. The second patient showed no signs of recurrence after 5 years of observation. There was no statistically significant relationship between lesion location and recurrence rate (*p* = 0.43), between lesion diameter and recurrence rate (*p* = 0.12), or between lid margin involvement and recurrence rate (*p* = 0.14).

## 4. Discussion

Among malignancies in humans, basal cell carcinoma (BCC) holds the distinction of being the most common, accounting for at least 80% of all non-melanoma skin cancers [39,40]. In general, basal cell carcinoma (BCC) displays a slow-growing nature and demonstrates a rare propensity for metastasis. Nonetheless, when left unattended or subjected to inadequate treatment, it can lead to substantial destruction of the local tissue and result in noticeable disfigurement [8]. While the mortality rate is less than 0.1%, BCC can cause severe morbidity [41]. Around 5% to 10% of all skin cancers occur in the periocular region, with eyelid skin cancers accounting for more than 90% of all ophthalmic cancers [9,10,42,43]. 

The goal of treatment of palpebral or periocular skin carcinomas is complete eradication of the tumor with minimal risk of recurrence, using the most effective treatment modality [22,23]. While options such as radiation, chemotherapy, and cryotherapy are occasionally indicated, excision is considered the treatment of choice for head and neck BCC due to its reliability and effectiveness [4,26,33,44,45,46,47,48].

To ensure effective management of BCC, it is essential to perform surgical excision with meticulous histological monitoring of clear surgical margins. Tragically, the recurrence rates for periocular BCCs that are resected without histological monitoring reach unacceptably high levels, reaching up to 67% [49,50,51,52,53,54].

In situations where immediate histological monitoring for clear margins is not available, it becomes imperative to ensure a wide margin of safety by excising a significant amount of clinically unaffected tissue surrounding the tumor. The primary objective is to minimize the potential for recurrence. To achieve comprehensive tumor removal and mitigate the risk of recurrence, it is generally advised to maintain a clinical safety margin of at least 4 mm [47,49,50,51,52,53,54]. However, by utilizing a clinical safety margin of 4 mm, the occurrence of recurrences in non-infiltrative primary periocular BCC is significantly decreased to an exceedingly unacceptable rate of 4.35% at the 5-year follow-up period [33,47,48,49,50,51,52,53,54].

Tragically, opting for wider margins has the unfortunate consequence of substantially enlarging the surgical tissue defect, thereby complicating the process of reconstruction. As a result, patients may experience increased morbidity, as the difficulty in achieving satisfactory reconstruction is heightened.

Whether a periocular BCC is primary or recurrent can have a serious impact on the outcome of surgery. When compared to primary BCC, recurrent BCC demonstrates more aggressive biological activity, which is associated with a poor prognosis and poor outcome [4]. In general, surgical excision of recurring tumors has a higher risk of inadequate excision, which might lead to recurrence of the disease.

It is critical to ensure histological surveillance of surgical margins in order to reduce the risk of recurrence and maintain unaffected tissue in the eyelid or periocular region. For this reason, two procedures have been developed: the traditional frozen section technique and the Mohs micrographic approach [22,54]. Mohs micrographic surgery (MMS) is a widely employed technique for the treatment of different types of skin malignancies, such as basal cell carcinoma (BCC). One of the primary benefits of MMS is its ability to preserve uninvolved tissue, which theoretically facilitates easier reconstruction. Consequently, it is regarded as an effective approach for excising BCC [4,22,23]. Excising periocular skin tumors with frozen section control (FSC) has been established as a reliable treatment approach for managing basal cell carcinoma (BCC) as an alternative to MMS [4]. During frozen section control, when evaluating surgical specimens, there are two methods for processing margin sections: perpendicular to the surgical margin and en face (or parallel) to the surgical margin. Traditionally, perpendicular sections are taken during tumor excision using frozen section control. However, a drawback of the perpendicular slices approach is that it does not examine the entire margin comprehensively. In contrast, the en face sectioning technique enables a comprehensive examination of the entire margin using a relatively small number of sections. To minimize the risk of misinterpreting the histological margin, it is essential to make thin sections. The tumor excision process utilizing CMC-FS involves various techniques, such as mapping the margins of the surgical specimen, tissue inking, precise and thin sectioning, clinical margin assessment, and en face sectioning with margins facing upwards. By employing CMC-FS, this approach effectively achieves oncological radicality comparable to that of Mohs surgeons.

Indeed, the similarities between CMC-FS and MMS outweigh their differences. Both techniques rely on the analysis of frozen sections to determine the extent of tumor excision and ensure complete removal. For tumors in the periocular region, both modalities typically require two separate surgical procedures. However, the key difference lies in the sequence of events. With Mohs surgery, the tumor is removed first, followed by histological analysis, and then the reconstruction performed by the oculoplastic surgeon. In contrast, with CMC-FS, the oculoplastic surgeon removes the tumor, and the reconstruction takes place as soon as the pathologist has completed the histological analysis of the tumor, effectively at the same time [2].

Another subtle contrast between the two techniques lies in the fact that while MMS strives to minimize the loss of normal tissue, irregularly trimmed margins may necessitate additional trimming by the reconstructive surgeon to achieve optimal alignment of the wound edges. However, when the same surgeon performing the frozen section surgical excision margin control is also responsible for the subsequent reconstruction of the periocular defect, they can strategically plan clear clinical margins with the reconstruction in mind. This means that it may not be necessary to sacrifice a larger amount of uninvolved tissue compared to what is ultimately sacrificed during MMS [26,33,47].

For the treatment of basal cell carcinoma using MMS, the recurrence rate ranges from 1% to 3% for primary tumors and 5.6% for recurrent tumors, encompassing all sites [55]. The cure rates observed in tumors involving the periocular region using the CMC-FS technique are comparable to those reported for MMS. In our experience, the CMC-FS technique has demonstrated cure rates of 1.8%, which aligns with the outcomes reported in the literature. While several published studies have reported 100% cure rates for tumors excised under frozen section control, it is important to note that none of these studies have matched the extensive series presented by Mohs [26,29,30,31,34,56]. Nevertheless, it is worth mentioning that these studies were conducted with relatively short follow-up periods and included a limited number of patients.

It is necessary that the follow-up of the studies is not less than 5 years to predict the actual degree of success in the surgical cure for BCC. Studies with a minimum follow-up of 5 years are few [25]. Our study holds notable strengths, including an extensive follow-up duration, a multidisciplinary evaluation involving anatomopathologists, and a substantial sample size comprising a significant number of patients. Nevertheless, it is crucial to recognize the inherent limitations of the study, namely its retrospective design and the fact that it was monocentric. Therefore, there is a need for a prospective multicenter study with an extended follow-up period to further validate and enhance the findings of our research.

## 5. Conclusions

Our observations revealed that the implementation of the complete margin control frozen section technique exhibited an eradication rate and subsequent recurrence rate comparable to that of the conventional Mohs micrographic surgery technique, widely regarded as the gold standard in the existing literature. This intriguing finding highlights the paradox that a faster and more expeditious technique can demonstrate a non-inferior profile when compared to the established benchmark in the field.

## Figures and Tables

**Table 1:** Patients’ clinical and pathological characteristics.

Characteristics	Data Set N = 111 (100%)
Age in years, mean (range)	69 (59–90)
Gender	
Male	47 (42.3%)
Female	64 (57.7%)
Previous treatment	
Yes	42 (37.8%)
No	69 (62.2%)
Anatomic localization	
Lower eyelid	81 (73%)
Upper eyelid	19 (17.1%)
Medial canthus	8 (7.2%)
Lateral canthus	3 (2.7%)
Mean maximum tumor diameter, cm (range, cm)	0.88 ± 0.99 (0.3–1.7)
Number of resection before all samples tested negative	
1	71 (64%)
2	24 (21.6%)
3	12 (10.8%)
4	3 (2.7%)
5	1 (0.9%)
Recurrence of BCC after treatment	
Yes	2 (1.8%)
No	109 (98.2%)

## Data Availability

Data are available on reasonable request by the corresponding authors.
